# Supplementary material for: Spontaneous Transition of Alkyl Carbocations to Unsaturated Vinyl-Type Carbocations in Organic Solutions
Source: Int J Mol Sci. 2023 Jan 16;24(2):1802. doi: 10.3390/ijms24021802 (PMC9860910; doi:10.3390/ijms24021802)
Supplement: Supplementary file 1 [file ijms-24-01802-s001.zip › ijms-2110310-supplementary.pdf]

SUPPORTING INFORMATION

# Spontaneous transition of alkyl carbocations to unsaturated vinyl-type carbocations in organic solutions

*Evgenii S. Stoyanov* *, Irina V. Stoyanova*

[a] N. N. Vorozhtsov Institute of Organic Chemistry, Siberian Branch of Russian Academy of Sciences, Akad. Lavrentiev Avenue 9, Novosibirsk 630090, Russia

* Corresponding author. E-mail address: evgenii@nioch.nsc.ru

**Experimental details**

The carborane acid, $H(CHB_{11}Cl_{11})$ (Figure S1), used for obtaining carbocation salts was prepared as described previously.[1] Methods for obtaining salts of cations *t*-$Bu^+$ and methylcyclopentyl$^+$ are given in refs. 2 and 3, respectively. Nonetheless, we found easier ways to prepare them. The t-$Bu^+(CHB_{11}Cl_{11})^-$ salt is easily obtained by wetting the acid powder with drops of liquid 1-chlorobutane followed by rinsing with a small volume of dichloromethane (DCM) and drying in a glove box with a water vapor content of <0.5 ppm. Salt methylcyclopentyl$^+(CHB_{11}Cl_{11})^-$ can be prepared as follows: the solid acid is dissolved in DCM to form a solution of $CH_3$-$CHB_{11}Cl_{11}$ and $(CH_2Cl)_2Cl^+(CHB_{11}Cl_{11})^-$ salts[4] followed by the addition of hexane. Hexane is protonated and converted into the methylcyclopentyl$^+$ cation, the salt of which precipitates as a white solid. It is separated, washed with DCM, and dried in a glove box. All the procedures were performed in a Vacuum Atmospheres Corp. glove box in an atmosphere of $N_2$ ($O_2$ and $H_2O$ concentrations <0.5 ppm).

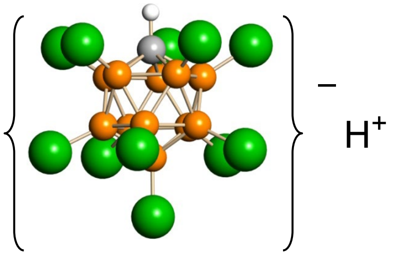


**Figure S1.** The superacid having icosahedral carborane anion $CHB_{11}Cl_{11}^-$

Crystals of $C_4$-carbocation salts were grown from a solution via slow evaporation of the solvent in the glove box or by keeping the solutions over $CCl_4$. X-ray structural analysis of the crystals was carried out by us earlier.[5-7]

IR spectra were recorded on a Shimadzu IRAffinity-1S spectrometer housed inside a glove box in either transmission or attenuated total reflectance (ATR) mode (400–4000 $cm^{-1}$). The IR data were processed in the GRAMMS/A1 (7.00) software from Thermo Fisher Scientific.

To record the IR spectra of the solutions, we used a cell with Si windows having 0.036 mm separation at the beam transmission point. To avoid interference effects, the cell configuration is wedge-shaped. The IR spectra of the solutions consist of overlapping spectra of the solute and solvent. To isolate a spectrum of a solute, the spectrum of the solvent was subtracted from the initial spectrum of the solution. Figure S2 shows a typical example of this procedure. Figures 1 and 2 in the main text present IR

spectra of the solutes in two frequency ranges: CH stretching vibrations and C=C stretching and CCH bending vibrations. The rest of the spectrum is not informative for our tasks.

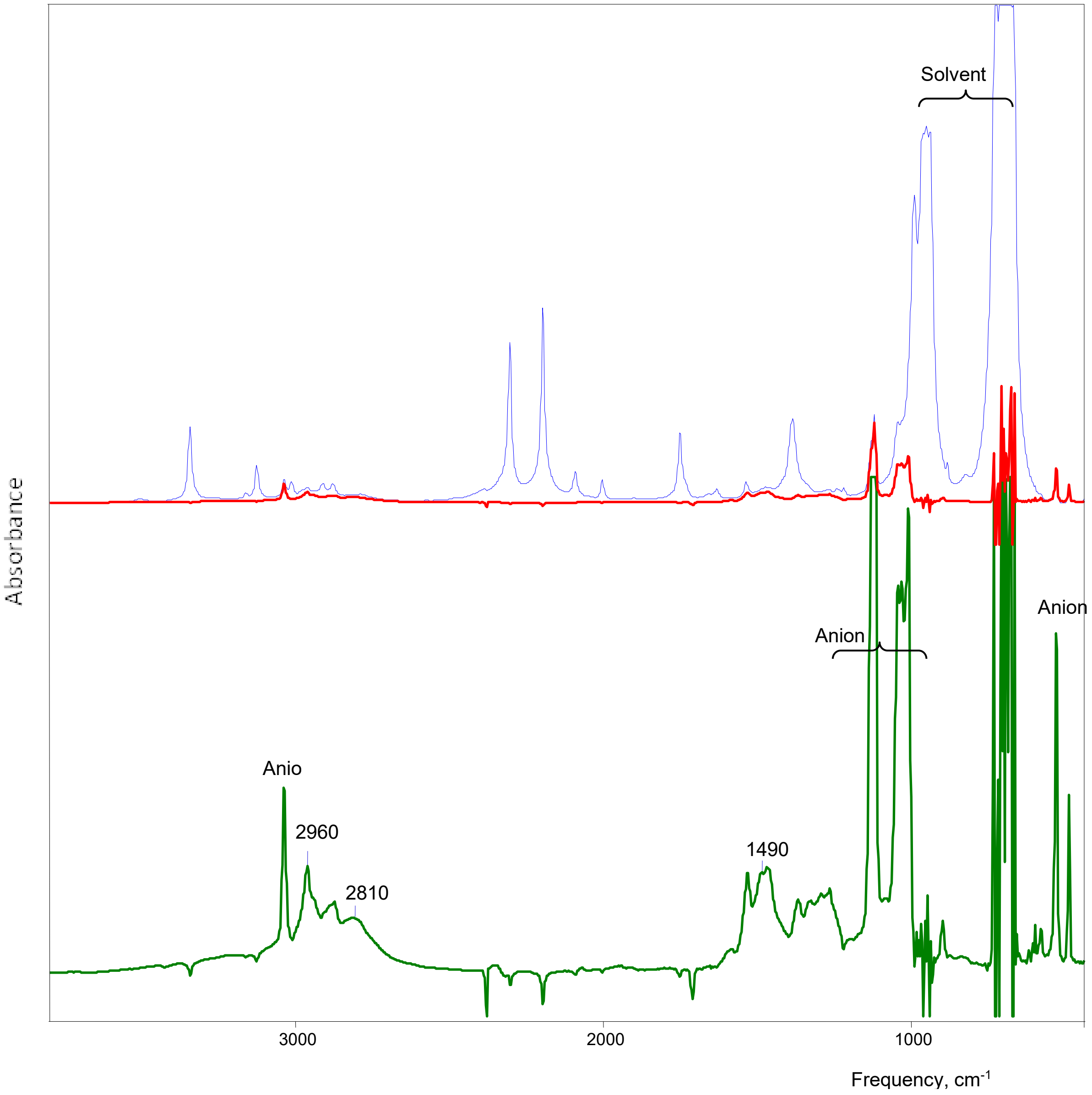


**Figure S2**. The IR spectrum of the *t*-Bu{$Cl_{11}$} solution in $CD_2Cl_2$, as recorded 2 min after preparation. Initial data are blue, and data with the subtracted spectrum of the solvent are red. The spectrum of the solute enhanced by a factor of 10 is green. In the main text, it is shown in Figure 1b, red.
